# Challenges encountered by family caregivers of prostate cancer patients in Cape Coast, Ghana: a descriptive phenomenological study

**DOI:** 10.1186/s12904-022-00993-6

**Published:** 2022-06-14

**Authors:** Benedicta Owoo, Jerry Paul Ninnoni, Evelyn Asamoah Ampofo, Abdul-Aziz Seidu

**Affiliations:** 1 School of Nursing and Midwifery, University of Cape Coast, Cape Coast, Ghana; 2 Centre for Gender and Advocacy, Takoradi Technical University, Takoradi, Ghana; 3 College of Public Health, Medical and Veterinary Sciences, James Cook University, Townsville, QLD 4811 Australia

**Keywords:** Family caregiver, Prostate cancer, Patients, Challenges, Cape Coast, Ghana, Nursing, Public health

## Abstract

**Background:**

In Ghana, prostate cancer is more prevalent than all other cancers, with a mortality rate of 75% partly due to late presentation to the health care facilities. Limited health services provision across the country and shortages of skilled nurses place a significant demand on family caregivers who are often ill-equipped for the caring role, resulting in many challenges. As they are expected to provide complex care at home with little resources, information, and support, the healthcare system rarely addresses the challenging needs of these family caregivers. This study explored the challenges encountered by family caregivers of people with prostate cancer.

**Methods:**

We conducted interviews using a descriptive phenomenological approach. Twelve family caregivers of prostate cancer patients were selected through a purposive sampling technique at the Cape Coast Teaching Hospital. All interviews were recorded, transcribed, and analysed using Colaizzi’s (1978) data analysis approach.

**Results:**

Three main themes were identified as critical challenges: education and training needs, resources and caregiver-nurse relationship issues. Under the three main themes emerged seven sub-themes that collectively hindered the family caregiver’s ability to meet the care demands. Sub-themes that emerged were; lack of preparedness towards the caring role, lack of knowledge about condition/ treatment, misconception about the condition, lack of accommodation facilities, financial constraints, poor communication, and poor staff attitude.

**Conclusion:**

Caregiving is associated with significant challenges that hinder the family caregiver’s ability to care for the patient effectively, further diminishing the caregiver’s quality of life and patient care. Therefore, healthcare professionals, especially nurses, should consider these challenges family caregivers face and take measures to obviate them through education, preparation and support.

**Supplementary Information:**

The online version contains supplementary material available at 10.1186/s12904-022-00993-6.

## Background

Prostate cancer is the second most frequently diagnosed cancer in men worldwide, accounting for 1.3 million new cases according to Bray et al. [1] and 358,989 deaths in 2018 [2]. In Africa, evidence suggests that the high incidence is due to low Prostate-specific antigen (PSA) testing, limited population-based cancer registries, and late presentation to health facilities [3]. The situation is, however, not different in Ghana, as people living with prostate cancer are mostly diagnosed late, resulting in a high (75%) mortality and low survival rate (17.7%) and reported as a leading cause of death among men [4]. The rate in Ghana reflects limited screening for cancers, poor access to healthcare facilities, and cultural beliefs [5, 6]. This has resulted in family members/ friends automatically assuming the role of a caregiver with little knowledge and support from health care professionals, especially nurses.

Family caregivers play an important role in the overall care of people with prostate cancer. The role of the family caregiver is increasingly being considered a valuable substitute for proper care; due to the current shift in healthcare delivery [6]. However, caring for a patient with cancer is generally viewed as one of the most stress-inducing caregiving challenges as it involves complex and sometimes unfamiliar procedures [7]. Given the intricate nature of the caregiving role, preparing the family caregiver for the task ahead is crucial. Family caregivers need knowledge and skills to perform the function effectively. Yet available literature suggests that many family caregivers receive little or no preparation at all [8, 9]. A paucity of evidence exists on the challenges family caregivers of patients with prostate cancer experience despite its high morbidity and mortality rate and the extensive involvement of family caregivers in caring for these patients.

Though, caring for a sick relative with a condition such as prostate cancer can be to some extent fulfilling. It may also be costly, especially in monetary terms. Cancer in the advanced stages may render the person jobless, yet, its management involves much money. The responsibility for ensuring that the patient goes through treatment rests on the family, in most cases, the family caregiver. Given et al. [10] reported that caregivers of cancer patients took loans, sold their homes, and used other financial resources to pay for the expensive cost of treatment that was not covered under health insurance. Others also complained of even going bankrupt due to the costly treatment [11].

Also, family caregivers have become part of a triad (where decision making during caring involves the health practitioner, patient and the family caregiver) of care, and effective caregiving requires that caregivers understand the course of the disease and the changing treatment goals. Knowledge about the condition and its management is one crucial area that has been neglected by healthcare providers, especially nurses [12]. Information on areas such as the cause, signs and symptoms, prevention, administration of medication at home to avoid medication errors, and identifying adverse effects are very vital details that need to be provided by healthcare professionals to improve the quality of care [10]. Sajjadi et al. [13] reported that health care professionals’ failure to give the required information about the patients’ disease and the caregivers’ health was a significant problem faced by family caregivers. Although most of these caregivers wanted to achieve more care-related information, this need remained unmet.

In Ghana, several studies have been conducted about the improving management of prostate cancer in respect to patient care with less emphasis given to the challenges faced by the family caregiver [14–18]. Although Ofori [19] addressed the experience of spouses of patients with prostate cancer, her study was limited in scope as other family members are involved in care delivery aside from spouses. Thus, identifying some of these challenges confronting the family caregivers and how it hinders their care can be a step towards resolving their problems. Therefore, this study explored the challenges encountered by the family caregiver of patients with prostate cancer in the Cape Coast Metropolis.

## Methods

### Study design and setting

The study was conducted at the Genito-urinary unit Out-Patient Department of the Cape Coast Teaching Hospital. This qualitative study is based on descriptive phenomenology, involving a purposive sampling of family members considered family caregivers by the patient. Our study was reported according to the consolidated criteria for reporting qualitative studies (COREQ) (Additional file 1).

### Participants recruitment

The participants included family caregivers of patients with prostate cancer. These provided the most assistance to the patients and were involved in the care for eight hours. Providing care for at least six months and above, and are aged ≥18 years. The study, however, excluded bereaved family caregivers who no longer cared for patients living with prostate cancer. The selection was made by purposive sampling from the Genito-urinary Out-Patient Clinic and the Male Surgical Unit of the Cape Coast Teaching Hospital (CCTH). The doctors recruited patients who met the inclusion criteria and directed them to the researcher in the consulting room. The patients were asked to identify their family caregivers, and their contact information was retrieved. Twelve participants were selected based on Creswell’s [20] recommendation of 5-25 participants. Also, emphasising saturation as suggested by Glaser and Strauss [21]. Data saturation was observed when no new information was obtained from the new participant’s [22]. None of the family caregivers declined.

### Data collection

Data collection was done using a pretested in-depth interview guide (see Additional file 2) to conduct face-to-face interviews (one on one) of twelve participants. No one else was present during the interview. This approach promoted confidentiality whiles allowing the participants to freely elaborate on their feelings, thoughts, and experience [20]. The lead researcher (who conducted the interviews) was also able to gain direct knowledge about participants’ experiences through broad and open-ended questions. The researcher kept a field note in which observations were recorded during the interview. The interviews lasted between 40 and 50 minutes. It took place in participants’ homes, within the hospital premises at the hospital snack bar/eatery, and outside some wards. Data collection continued until data saturation was reached. The data collection lasted between March and May 2019. All interviews were audio-recorded and transcribed verbatim concurrently.

### Ethical issues

The study was approved by the Institutional Review Board (IRB) of the University of Cape Coast (UCC) (UCCIRB/CHAS/2018/24) and the ethical committee of the CCTH research unit (CCTH/RDS/2019/41). Each participant was given a consent form to read and sign/thumbprint, those who did not have the literacy ability were assisted. All misunderstandings were clarified about days/week before the interview.

### Data analyses

This study conducted data analysis concurrently with data collection using Colaizzi’s [23] data analysis approach by three researchers (BO, JPN and EAA). Following closely the data analysis approach proposed by Colaizzi [23], the first step involves the researcher reading all the participants’ verbatim transcripts to acquire their feel. Step two involved extracting significant statements deemed relevant to the study. Step three is followed by formulating meanings from the relevant messages removed earlier [24]. In step four, the researchers then grouped the identified meanings into common themes across all accounts. The researchers wrote a full and inclusive description of the phenomenon in step five, incorporating all the themes produced at step four*.* Step six typically involved a return to research participants for member-checking or validation of created profiles of their experiences and verification. Finally, step seven incorporated any new or pertinent data obtained into the final study. However, no further information came up.

#### Trustworthiness

The four main criteria proposed by Lincoln and Guba [25] were followed to guarantee the study’s trustworthiness, and Bracketing was ensured. Lincoln and Guba [25] proposed the four main criteria: credibility, which ensured that participants maintained contact to verify if the study’s findings were true to their experiences. By using member checking, “acquired by getting agreement from the participants on the emerged results through phone calls [20]. Transferability was also performed by recruiting nominated samples, Dependability by keeping an audit trail. Comfirmability was established by verifying results with participants and using their own words in generating the description of their experiences.

Bracketing involves putting aside repertoires of knowledge, beliefs, values, and experience as a practising nurse to describe the participants’ life experiences accurately [26]. Also, means holding in abeyance those elements that define the limits of an experience when the nurse is uncovering a phenomenon about which he/she knows a great deal [27]. Bracketing was ensured by documenting the researcher’s preconceptions before the onset of the study through a reflective diary, thus eradicating any biases inherent in the researcher’s beliefs and attitudes [20]. The lead researcher again promoted bracketing by participants because she realised that the knowledge of her being a nurse (in a different facility outside of Cape Coast which she had disclosed to the participants before the commencement of data collection) made it difficult for some of the participants to frankly express their feelings/opinion; believing that their responses may affect the care that was rendered to their patients [28]. Also, to ensure that the data collected is not distorted or filtered, Colaizzi’s method of data analysis was employed to validate results by returning to study participants.

## Results

The demographic characteristics of participants are presented in Table 1.

**Table 1:** Participants demographic characteristics

Pseudonyms	Marital status	Religion	Educational level	Relationship to patient	Duration of Care	Occupation
Love	Married	Christian	Junior High Sch	Father	2 years	Seamstress
Peace	Not Married	Christian	Tertiary	Father	1 year	Secretary
Grace	Married	Christian	Tertiary	Father	1 year	Nurse(Awaiting postings)
Joy	Married	Christian	Primary	Husband	1 year	Trader
Mercy	Married	Christian	Tertiary	Husband	2 year	Retired
Forgive	Married	Christian	Tertiary	Husband	1 year	Retired
Favour	Married	Christian	Senior High Sch	Father	1 year	Trader
Humble	Married	Christian	Illiterate	Husband	1 year	Farmer
Passion	Married	Christian	Illiterate	Husband	1 year	Farmer
Kindness	Married	Christian	Tertiary	Husband	2 years	Trader
Hope	Not Married	Christian	Tertiary	Father	6 months	Teacher
Faith	Married	Christian	Illiterate	Father	1 year	Trader

### Themes from the study

**Fig. 1:**
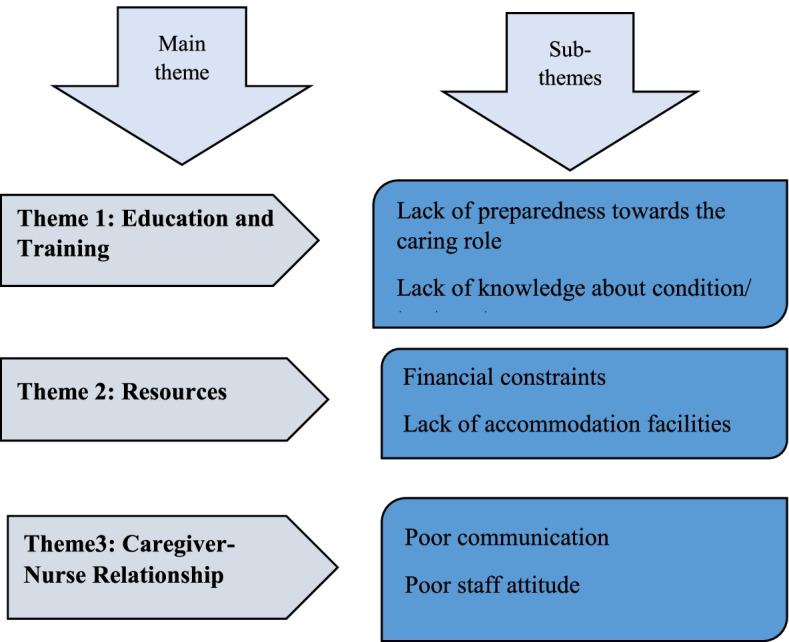
Themes and Sub-themes

Family caregivers of prostate cancer patients in the Cape Coast metropolis face challenges that impact their quality of life and well-being. The main themes and subthemes that emerged from the study are presented in Fig. 1.

### Theme 1: education and training needs

This theme describes caregivers’ education and training needs that impact their caring roles. Emerging subthemes include lack of preparedness towards the caring role, lack of knowledge of the condition and misconceptions about the condition.

### Lack of preparedness towards caring role

Preparedness in this study has to do with a caregiver’s perceived readiness for the caregiving role, which encompasses multiple domains, including the provision of physical care and emotional support. Preparing the family caregiver for the caring role is an important area that needs to be addressed by health professionals, especially nurses. Yet too often, these family caregivers are poorly prepared for this vital but challenging role that takes a toll on them, by extension, the patient. Participants in the present study reported not being asked by healthcare professionals what they needed to care for the patient. They stated

“*No one educated us on how to take care of him at home”,* ***Grace***

“No one took the time to explain to us how to care for him at home.” ***Mercy***

*“They educated us on how to administer the medications. But nothing else”* ***Humble***

### Lack of knowledge about condition/treatment

Caregivers have become part of the triad of care. Effective caregiving and decision-making require that they understand the course of disease/ treatment and resources available to them. Their ability to care for their patients may be hampered by their lack of understanding of the disease and treatment. Some participants reported a lack of knowledge about the condition and treatment, resulting in the patient going through much pain. Others also reported confusion due to inadequate education on the condition and treatment. At the same time, others reported having received adequate education on the condition and treatment. All participants again reported not being informed about resources available.

Some participants’ lack of knowledge about the condition and treatment confused them. For example, a participant had this to share:

*“I suggest that in situations of this sort, the patient and relations should be educated on what is going on. For instance, we were told he had prostate cancer when we came. Today too, we have just been told that he will have an orchidectomy…Laughing. No one is explaining things to us. They tell you these big words without breaking them down for you to understand”* ***Grace***

Some participants also indicated that lack of education on condition and treatment resulted in their patients going through much pain. The following quote illustrates this:

*“…One more thing is that initially, we were not told that the catheter must be changed after some time. No one informed us about that, so he had it on for long, resulting in pain. The catheter stayed in for over a month and two weeks. When it should have been in there for just three weeks and then changed at the hospital”* ***Peace***

Nonetheless, some participants indicated that they received education on the condition and treatment. Mercy had this to share:

*“The doctor explained everything about the condition before surgery. The things he will need for the surgery were communicated to us. Also, upon discharge, we were told how to administer the medications at home”* ***Mercy***

While some participants received an education, others read about the condition from the internet.

*“No one, but what I do is I read around the condition and also on the medications from the internet. Apart from consulting our family doctor friend”* ***Hope***

It was also revealed that all the 12 participants reported not being informed about resources available to them by healthcare personnel such as counselling services. They stated that

*“I have never been to a counsellor before, wasn't aware they existed in this hospital”* ***Favour***

Misconception about the condition.

One of the challenges that family caregivers encounter in caring for their relations with prostate cancer is people’s beliefs and misconceptions about the condition. It has become clear that cancer-related stigma and myths are significant problems that must be addressed. Findings from this study show evidence of myths associated with cancer, such as the belief that cancer is a fatal and contagious disease. Others also believe that one may contract it due to immoral behaviour or a punishment from God or enemies. Family caregivers reported that these misconceptions lead to fear/panic and a decrease in the support network.

*“People say it is a condition that has no cure, so I was scared when I first heard he has cancer”* ***Love***

*“Eeeii… in my place, when you hear cancer, it means that the person is going to die. You are waiting to die. Others don't even want to hear the word at all. Some also say it is infectious. So, after telling them, you will notice that they begin to isolate themselves from you and the patient”* ***Grace***

Participant believes that cancer may result from something terrible done in the past/ evil done to others

*“Anything may be the cause. Currently, the world is being ruled by the devil so that anything can happen. Someone may contract a condition due to something done in the past. Others, from their enemies”* ***Forgive***

### Theme 2: resources

Limited resources were reported as challenges to caregivers roles. Relevant themes that emerged include; financial constraints and lack of accommodation.

### Financial constraint

All participants admitted having financial difficulties. Some mentioned that it resulted in a delay in treatment, causing the patient to go through so much pain. Others had to try herbal preparation, which made the condition worse. One participant mentioned that the government must support because most patients were retirees.

*“We were required to deposit Ghc1000 that same day so that the surgery can be done the following day, but unfortunately for us, we could not raise the amount, so we had to go back and come after we raised the money” Love*

*“In the beginning, I took a loan because I had spent all my money on medical expenses”* ***Peace***

Participant complained of financial difficulties and also lack of support from the government

*“Another issue has to do with money; the little money we raise must go into this sickness. Either we are buying medications or paying for hospital bills and lab tests. We are both retired. Besides, as pensioners, some medications must be free; we have served the country our entire life. The national health does not cover most of the investigations. At our age, we don't have money; the government must do something about it”* ***Mercy****.*

### Lack of accommodation facilities

Some participants said that the hospital where their patients received treatment was far from their home, which was tiring for them to commute to and from the hospital when their patients were on admission. It resulted in financial difficulties from the high expenses on transportation to and from the hospital. Besides, the hospital did not provide caregivers with any place to stay, resulting in them sleeping outside the wards, on the floor and being exposed to poor conditions and mosquito bites. Some caregivers stated:

*“My home is quite far, so if I always have to go and return, it will be costly. Again, the hospital has not provided a place for us; we sleep outside on the floor and are exposed to mosquito bites”* ***Humble***

*“Even me...I would have stayed in a place where I could spend the night. The hospital has no place for caregivers who have their patients on admission; we have to sleep outside on the floor, yet, if there is something to be done for the patient and you are not around, it won't be done until you return”* ***Love***

### Theme 3: caregiver-nurse relationship

A good nurse-caregiver relationship has therapeutic benefits; however, participants reported concerns. Sub-themes that emerged include poor communication between caregivers and nurses and poor attitudes towards caregivers.

### Poor communication

A primary role of cancer family caregivers involves interacting with various providers and professionals on behalf of their loved ones. Lack of effective communication between healthcare personnel and family caregivers is an important issue, mainly because it can lead to errors and poor patient outcomes. Findings from the study show that patients went through avoidable pains due to ineffective communication. Also, it resulted in an unnecessary waste of resources and a feeling of inadequacy.

Participants reported poor communication as follows:

*“By the grace of God, we were able to raise money for the surgery. However, the date scheduled for the surgery was cancelled after all preparations were made; we were then asked to send my dad home. The cancellation was not communicated to any of us, yet he was discharged to be brought back a week later. We still had to pay for the discharge.”* ***Love***

*“Sometimes I don't even know what to do to help him because no one told me anything”* ***Passion***

As if the challenges encountered by these family caregivers are not enough, even within the health care environment are faced with even more difficulties that further impact their role. Family caregivers encounter many challenges within the hospital environment that affect the care they provide to their patients. Participants shared their experience on the challenges they faced within the hospital environment.

### Poor staff attitude

Providing quality care involves doing the right thing at the right time and improving the health outcome for both patients, their family caregivers, and the community at large. It is also essential to ensure a welcoming atmosphere within the healthcare environment. However, this is mostly not the situation. Participants complained of poor staff attitude and unfavourable conditions within the healthcare environment, negatively affecting their role. Some caregivers complained that they had to wait long hours at the Out Patient Department (OPD) before the clinic started; others complained of poor staff attitude, making it impossible to approach them. One participant also pleaded that conditions be improved concerning the neglect of care when family caregivers are not around.

A wife of one of the patients’ stated:

*“When we visit the hospital, there is too much waiting time. We come very early, but the clinic does not start early. They must try and do something about it for us. Because we are already exhausted, and some of the patients are also in pain and very sick”* ***Mercy***

Another participant also mentioned

*“Also, to the hospital staff, especially the nurses and doctors, I know with every work there is time to start. They must report at the right time because patients come to the hospital and wait long before seeing. They must also watch how they relate to the patients and their relatives, especially nurses. Their attitude makes it difficult for us even to ask them for anything” Forgive*

*"Another issue has to do with some nurses; some are very disrespectful. They must change their attitude towards caregivers and patients. Because sometimes we want to ask them to clarify something, but we are not able to, for fear of being embarrassed”* ***Kindness***

Participants complained of staff neglecting patients in the absence of family caregivers.

*“Please, I would like to plead with the ward staff that if there is anything that needs to be done for our patients about their treatment, they should do it so that when the relatives come around, they can pay for them, rather than neglect the patients when the relatives have been sent out of the unit. It is very frustrating”* ***Faith.***

Participant complained about lack of support from hospital staff

*"Mostly when we come, I have to do all the errands, go here, go there, and also wheel him around all by myself, so this time my son was home, so we came with him. Formally, some orderlies help relatives with wheeling patients, but now they don't do it anymore. You can imagine that if I had not come along with my son what I was going to do because I couldn't have wheeled him around in this state"* ***Forgive***

Participant, however, commended the nurses for their excellent work

*She stated, “aww, the nurses did very well; they treated the situation with urgency. Immediately, they called the doctor and put him in bed and started with treatment”* ***Mercy***

## Discussion

This study demonstrated a range of challenges experienced by prostate cancer family caregivers in the Cape Coast metropolis. Family caregivers are faced with diverse challenges as they take up the role of caregiving. The findings revealed major challenges such as lack of preparedness towards the caring role, lack of knowledge about condition and treatment, misconception about the condition, financial constraints. Poor staff attitude and poor communication concerns were also reported. However, all other findings under these themes are discussed below.

### Education and training

#### Lack of preparedness towards caregiving role

Lack of preparedness for caregiving was a significant concern among all the participants. The family caregivers reported receiving no preparation for caregiving and hence lacked the knowledge and skills to play the role effectively. Like the findings reported in this study, Mazanec et al. [8] stated that family caregivers often feel unprepared to provide care, have inadequate knowledge to deliver proper maintenance, and receive little guidance from the formal health care providers. Besides, the report from the current study participants showed that they were more than willing to take on the responsibility of patient care. However, preordained financial and psychological burdens as most caregivers considered the assistance they provided “Giving back”. Within the typical Ghanaian setting, it is the social responsibility of the female spouses/ children to take care of their sick husbands/fathers. Yet, they lacked the skills and knowledge required to take up the role.

#### Lack of knowledge about the condition and its management

Almost all the participants cited a lack of knowledge about the condition and its management as an unmet need. It is an area that healthcare providers have neglected. Most of the participants reported having received little to no knowledge of the condition and its management. It was reported that the number of times to administer the medication was communicated to them. However, no education was given on the side/ adverse effects of the medicines, identifying these side effects, and even the measures to take at home before bringing the patient back to the health facility if necessary. Etemadifar et al. [13] and Sajjadi et al. [13, 29] affirm this assertion that caregivers reported the failure of specialists to give the required personal information and specialist knowledge about the patients’ disease the caregivers’ health as a major problem that remained unmet. Travis et al. [30] further reported that the lack of knowledge on the condition and its management resulted in medication errors. Therefore, it is imperative that as health practitioners, the required information is provided to reduce the uncertainty and stress among these family caregivers, thus promoting patient’s quality care.

#### Misconception about the condition

Again the findings of this study disclosed that the majority of the participants expressed many misconceptions about the condition. This indicates that culture plays a significant role in how caregivers perceive and go about caregiving. Some participants reported that cancer might be contracted due to immoral behaviour or punishment from God, which is consistent with the finding of a study by Kuan Lee Wai, [31]. Others also believe it is a punishment from one’s enemies for mistreating them; this mirrors Lui and Ip [32]. Another finding reported by participants was that cancer is contagious and fatal, confirmed by Lee and Bell [33]. These beliefs resulted in fear/ panic and further decreased the support network [34]. A single relevant perspective that can be drawn from this finding is that the people within the context of the study need to be well educated about prostate cancer/cancer in general. However, in contrast to the work of Lee [35], no association was reported between caregiver tension and the diagnosis of cancer.

### Resources

#### Financial constraint

Financial constraint was another problem that came up. The majority of the participants complained of financial difficulties. Some had to depend on their children, friends, and other family relatives, while others for loans. Some participants had to use up their savings and retirement benefits. Some of the patients /participants had to try herbal preparation due to the high cost of treatment. And others went bankrupt due to the expenses involved in managing this condition. This finding is echoed by Given et al. [10], who reported in their study that caregivers of cancer patients took loans, sold their homes, and used other financial resources to pay for the expensive cost of treatment that was not covered under health insurance and even went bankrupt [11]. The finding also revealed that most of these caregivers had lost their livelihood due to the increased physical and financial burden of the role [36, 37]. This financial challenge appears to reflect the economic status of the study setting as Cape Coast is one of the poorest regions in Ghana. However, in contrast with the present study’s findings, Mosher et al. [38] revealed that complementary and alternative medicine was viewed to be more favourable/ effective. Instead, participants in the present study worsened their condition, making the caregivers’ role more difficult when they opted for herbal preparation. To add to the existing knowledge report from the current study revealed that the use of the herbal practice to manage prostate cancer worsened the patient’s condition, further causing more distress/stress to the caregiver.

#### Lack of accommodation facility

Finding has revealed that these unpaid caregivers encounter problems at home. The healthcare environment is also another place that may negatively contribute to the caregiving role, resulting in more stress and distress. Lack of accommodation facilities was a problem within the healthcare environment. The majority of the participants narrated how they slept on floors outside the ward in the open. These family caregivers were exposed to harsh weather conditions and mosquito bites due to a lack of accommodation facilities. Others also mentioned that they had to stay because their home is far from the hospital. Communing to and from the facility was financially draining, coupled with their loss of livelihood and expensive medical costs making life very difficult. This finding is consistent with the work of Sadigh et al. [39], who studied the Economic and Social Impact of Informal Caregivers at Mulago National Referral Hospital, Kampala, Uganda revealing that Ninety per cent of informal caregivers stayed at the hospital for 11 days on average, with a range of 1-60 days. While staying at the hospital, 89% slept on the hospital floor. Three per cent returned home each evening to rest; 2 % slept in the prison barracks where members of the police force stayed, 1 % slept in Bwaise, an urban slum, and the other 1 % shared the hospital bed with the patient. This finding indicates that it is essential that hospitals provide a resting place for these family caregivers especially considering a referral point hospital like the Cape Coast teaching hospital.

### Caregiver -nurse relationship

#### Poor staff attitude/poor communication

Poor staff attitude/communication was also reported as a challenge encountered within the health care environment. Some participants reported waiting long hours at the Out Patient Department (OPD) before the clinic started. Others also complained of poor staff attitude, making it difficult for them to be approached, and neglected patient care when a family caregiver is not around. Of concern in these findings were caregiver reports of a poor attitude. According to Delicado Users et al. [40], nurses’ attitudes and activities with caregivers are influenced by lack of time, hospital workload, and organisation. Although these reports were few and most caregivers tolerated such behaviour, it made it difficult for coping/information-seeking. It is, therefore, necessary that healthcare providers ensure better behaviour to improve upon customer service and patient/caregiver satisfaction through the organisation of workshops for its staff. Efforts must also be made to increase the staffing numbers.

### Implications of the study findings for nurses and the health system

#### Nursing practice

For ages, health care providers and, most importantly, nurses have concentrated on providing care for patients while neglecting the needs and concerns of the family members/loved ones who cared for these patients at home. The findings of this study have brought to light that these family caregivers are hidden patients themselves, hence as nurses, we must perceive them so that care can be extended to them. Similarly, nurses must endeavour to assess the family caregiver’s needs to help provide the care/assistance required. To achieve this, the nurse must develop a professional relationship with the patients and their family caregivers, especially those who build trust and emotional support to help caregivers feel more comfortable and willing to express their feelings. Furthermore, family caregivers need preparation to meet the demands of their new responsibilities. Nurses must, therefore, focus on adequately preparing the caregivers (with the knowledge, information, skills, and resources) to assume the caregiver role. Likewise, there is the need to provide both patients and family caregivers/communities with information on the causes, signs/symptoms, and management of cancer (prostate cancer). This will help a long way to clear these family caregivers’ misconceptions about the condition.

#### Nursing research

The present study’s findings brought to bear that culture played a significant role in how family caregivers perceived their position and even how the care is rendered to these patients. Therefore, there is a need for further studies on the role of culture in the experiences of prostate cancer family caregivers among a multicultural group to help establish findings per other cultures. Also, further research is required in the area of preparedness of family caregivers of prostate cancer patients towards the caregiving role.

#### Health system

Based on the study findings, the health system, especially referral point hospitals, must make available accommodation facilities in their small way for the family caregivers. Considering this can, in the long term, generate revenue for the facility. Also, the waiting time at the OPD must be checked since it has been reported as a contributing factor to caregiver stress. Absorbing the cost of screening and treating prostate cancer under the national health insurance scheme will greatly ease caregiver financial burden. There is also the need to increase the staffing numbers in health care facilities.

#### Limitations of the study

The focus of the study was on family caregivers of prostate cancer patients. However, it is essential to acknowledge challenges, especially with the healthcare personnel and the patients themselves. Thus, the findings may not necessarily represent caregivers of patients with other health conditions. Nevertheless, the researcher was not interested in generalising findings instead of making meaning into experiences shared by participants. The findings also revealed that all participants were Christians (not purposeful); there could be a limitation to the non-Christian population. Also, culture played a significant role in caring for these family caregivers in the current study context. However, other groups within the country may present a different picture.

## Conclusion

Caregiving is associated with significant challenges that hinder the family caregiver’s ability to care for these patients effectively. This study has brought to light some of the difficulties encountered by this group. Therefore, as healthcare professionals, especially nurses, it is essential that these family caregivers are equipped with the knowledge, skills, resources, and supports to provide care and prepare adequately for the new role while maintaining their health and well-being and delivering cancer care for their patients.

## Supplementary Information

**Additional file 1.**

**Additional file 2.**

## Data Availability

The datasets used and analysed during the current study are available from the corresponding author on reasonable request.
