# Supplementary material for: Challenges encountered by family caregivers of prostate cancer patients in Cape Coast, Ghana: a descriptive phenomenological study
Source: BMC Palliat Care. 2022 Jun 14;21:108. doi: 10.1186/s12904-022-00993-6 (PMC9195311; doi:10.1186/s12904-022-00993-6)
Supplement: Supplementary file 1 — Additional file 1. [file 12904_2022_993_MOESM1_ESM.docx]

**Additional file 1: COREQ checklist**

**Consolidated criteria for reporting qualitative studies (COREQ): a 32-item checklist**

| No. Item | Guide questions/description | Reported on Page # |
| --- | --- | --- |
| Domain 1: Research team and reﬂexivity |  |  |
| *Personal Characteristics* |  |  |
| 1. Interviewer/facilitator | Which author/s conducted the interview or focus group? | Page 5 |
| 2. Credentials | What were the researcher’s credentials? E.g. PhD, MD | Page 1 |
| 3. Occupation | What was their occupation at the time of the study? | Page 1 |
| 4. Gender | Was the researcher male or female? | Page 1 |
| 5. Experience and training | What experience or training did the researcher have? | Page 1 |
| *Relationship with participants* |  |  |
| 6. Relationship established | Was a relationship established prior to study commencement? | Page 5 |
| 7. Participant knowledge of the interviewer | What did the participants know about the researcher? E.g. personal goals, reasons for doing the research | Page 7 |
| 8. Interviewer characteristics | What characteristics were reported about the interviewer/facilitator? E.g. Bias, assumptions, reasons and interests in the research topic | Page 7 |
| Domain 2: study design |  |  |
| *Theoretical framework* |  |  |
| 9. Methodological orientation and Theory | What methodological orientation was stated to underpin the study? E.g. grounded theory, discourse analysis, ethnography, phenomenology, content analysis | Page 1 and 5 |
| *Participant selection* |  |  |
| 10. Sampling | How were the participants selected? E.g. purposive, convenience, consecutive, snowball | Page 5 |
| 11. Method of approach | How were participants approached? E.g. face-to-face, telephone, mail, email | Page 5 |
| 12. Sample size | How many participants were in the study? | Page 5 |
| 13. Non-participation | How many people refused to participate or dropped out? Reasons? | 5 |
| *Setting* |  |  |
| 14. The setting of data collection | Where was the data collected? E.g. home, clinic, workplace | Page 5  . |
| 15. Presence of non-participants | Was anyone else present besides the participants and researchers? | Page 5 |
| 16. Description of sample | What are the important characteristics of the sample? E.g. demographic data, date | Page 8 |
| *Data collection* |  |  |
| 17. Interview guide | Were questions, prompts, guides provided by the authors? Was it pilot-tested? | Page 5 |
| 18. Repeat interviews | Was repeat interviews carried out? If yes, how many? | No |
| 19. Audio/visual recording | Did the research use an audio or visual recording to collect the data? | Page 6 |
| 20. Fieldnotes | Were ﬁeld notes made during and/or after the interview or focus group? | Page 5 |
| 21. Duration | What was the duration of the interviews or focus group? | Page 5 |
| 22. Data saturation | Was the data saturation discussed? | Page 5 and 6 |
| 23. Transcripts returned | Were transcripts returned to participants for comment and/or correction? | Page 6 |
| Domain 3: analysis and ﬁndings |  |  |
| *Data analysis* |  |  |
| 24. Number of data coders | How many data coders coded the data? | Page 6 |
| 25. Description of the coding tree | Did authors provide a description of the coding tree? | Page 6 |
| 26. Derivation of themes | Were themes identiﬁed in advance or derived from the data? | Page 9 |
| 27. Software | What software, if applicable, was used to manage the data? | N/A |
| 28. Participant checking | Did participants provide feedback on the ﬁndings? | 6 |
| *Reporting* |  |  |
| 29. Quotations presented | Were participant quotations presented to illustrate the themes/ﬁndings? Was each quotation identiﬁed? E.g. participant number | Page 10 to 16 |
| 30. Data and ﬁndings consistent | Was there consistency between the data presented and the ﬁndings? | Yes, there was.  Page 10-20 |
| 31. Clarity of major themes | Were major themes clearly presented in the ﬁndings? | Yes. They were.  From page 9- 14 |
| 32. Clarity of minor themes | Is there a description of diverse cases or discussion of minor themes? | Discussion of major and minor themes  From page 9 to 16 |
