# Supplementary material for: Challenges encountered by family caregivers of prostate cancer patients in Cape Coast, Ghana: a descriptive phenomenological study
Source: BMC Palliat Care. 2022 Jun 14;21:108. doi: 10.1186/s12904-022-00993-6 (PMC9195311; doi:10.1186/s12904-022-00993-6)
Supplement: Supplementary file 2 — Additional file 2. [file 12904_2022_993_MOESM2_ESM.docx]

**Additional file 2****: DATA COLLECTION INSTRUMENT**

**Interview Guide**

**SECTION A**

1. **Demographic Information**

Identification (ID) Code ………………….

Age

Sex

Nationality

Highest educational level

Occupation

Religion

What is your relationship with the patient?

**SECTION B**

1. **What are the experiences of caregivers caring for people with prostate cancer?**

How long has your relationship been living with the condition?

How long have you been providing care for the patient?

Please kindly tell me about your relation’s condition

What exactly do you usually do for him daily?

1. **What are the challenges to caring for patients with prostate cancer?**
2. Please, can you tell me what comes to your mind when you hear that someone has cancer?
3. What does cancer mean to you and your family?
4. How do people interpret cancer here on Cape Coast?
5. When you first learned that your relationship had been diagnosed with cancer, what was your reaction?
6. How would you describe the preparation given to you by the nurses/healthcare team before assuming the caring role?

Probe:

Did you receive any education on the condition?

Did you receive any instruction on how to manage pain?

Did you receive any education on how to administer medications?

Did you receive any coaching on identifying and addressing the adverse effects of medications?

1. Please, in terms of finances, how would you describe your funding when it comes to providing care.

Probe:

How do you support yourself financially, e.g. Loans, support, donation

**SECTION C**

1. What resources have you used so far to help you get through this situation?
2. Would you like to share a saying that describes this experience?
3. Please, is there any other thing you would like me to know?
